# Targeted Biologic Therapies in Severe Asthma: Mechanisms, Biomarkers, and Clinical Applications

**DOI:** 10.3390/ph18071021

**Published:** 2025-07-10

**Authors:** Renata Maria Văruț, Dop Dalia, Kristina Radivojevic, Diana Maria Trasca, George-Alin Stoica, Niculescu Stefan Adrian, Niculescu Elena Carmen, Cristina Elena Singer

**Affiliations:** 1 Research Methodology Department, Faculty of Pharmacy, University of Medicine and Pharmacy of Craiova, 200349 Craiova, Romania; renata.varut@umfcv.ro (R.M.V.); kristinaradivojevic03@gmail.com (K.R.); 2 Department of Mother and Baby, University of Medicine and Pharmacy of Craiova, 200349 Craiova, Romania; dalia.dop@umfcv.ro (D.D.); drcarmen88@yahoo.com (N.E.C.); cristina.singer@umfcv.ro (C.E.S.); 3 Department of Internal Medicine, University of Medicine and Pharmacy of Craiova, 200349 Craiova, Romania; 4 Department of Pediatric Surgery, Faculty of Medicine, University of Medicine and Pharmacy of Craiova, 200349 Craiova, Romania; 5 Department of Orthopedics, University of Medicine and Pharmacy Craiova, 200349 Craiova, Romania; niculescustefan94@gmail.com

**Keywords:** airway inflammation, cytokines, type 2 inflammation, asthma exacerbations

## Abstract

Asthma represents a heterogeneous disorder characterized by a dynamic balance between pro-inflammatory and anti-inflammatory forces, with allergic sensitization contributing substantially to airway hyperresponsiveness and remodeling. Central to its pathogenesis are cytokines such as IL-4, IL-5, IL-13, IL-17, and IL-33, which drive recruitment of eosinophils, neutrophils, and other effector cells, thereby precipitating episodic exacerbations in response to viral and environmental triggers. Conventional biomarkers, including blood and sputum eosinophil counts, IgE levels, and fractional exhaled nitric oxide, facilitate phenotypic classification and guide the emerging biologic era. Monoclonal antibodies targeting IgE (omalizumab) and IL-5 (mepolizumab, benralizumab, reslizumab, depemokimab) have demonstrated the ability to reduce exacerbation frequency and improve lung function, with newer agents such as depemokimab offering extended dosing intervals. Itepekimab, an anti-IL-33 antibody, effectively engages its target and mitigates tissue eosinophilia, while CM310-stapokibart, tralokinumab, and lebrikizumab inhibit IL-4/IL-13 signaling with variable efficacy depending on patient biomarkers. Comparative analyses of these biologics, encompassing affinity, dosing regimens, and trial outcomes, underscore the imperative of personalized therapy to optimize disease control in severe asthma.

## 1. Introduction

Asthma affects roughly 358 million individuals worldwide, and its occurrence varies widely by region [1]. Rates tend to be higher in cities and among people exposed to known risk factors, such as allergens, tobacco smoke, and air pollution. In many high-income countries, new cases have leveled off after decades of rise; by contrast, low- and middle-income nations are seeing rapid increases in asthma prevalence, likely fueled by worsening fossil fuel emissions and shifts toward Western lifestyles. Limited access to precise diagnostic tools and uniform treatment protocols in these regions further amplifies the burden on patients, families, and healthcare systems [2].

The hallmark of asthma is expiratory airflow obstruction, which is generally reversible yet stems from narrowing of the airway lumen. Chronic inflammation of the airway wall, driven by infiltrating and activated immune cells (eosinophils, neutrophils, lymphocytes, dendritic cells, innate lymphoid cells, and mast cells), triggers bronchial hyperresponsiveness, mucus overproduction, and structural remodeling of the airways.

Clinically, asthma presents with symptoms such as shortness of breath, coughing, wheezing, chest tightness, progressive loss of lung function, frequent exacerbations, and variable disease severity. These diverse features reflect multiple underlying mechanisms in which interactions between structural cells and immune elements give rise to the pathological traits of asthma. Genetic differences and environmental exposures further shape each patient’s profile, leading to considerable heterogeneity in symptoms and biomarker patterns.

The 2023–2024 GINA updates classify several common asthma phenotypes: allergic asthma, nonallergic asthma, adult-onset (late-onset) asthma, asthma with persistent airflow limitation, and obesity-associated asthma [3]. In parallel, animal studies—particularly murine models of allergic airway inflammation—have confirmed a central role for T helper 2 (TH2) cells in driving airway inflammation and hyperreactivity [4,5]. TH2 cytokines mediate key asthma characteristics: IL-5 for tissue eosinophilia, IL-13 for bronchial hyperresponsiveness, and IL-4 plus IL-13 for goblet cell metaplasia [6].

More recently, other innate immune populations—mast cells, basophils, group 2 ILCs, “M2” macrophages activated by IL-4/IL-13, and a subset of IL-4-secreting NK/NKT cells—have been shown to contribute to type 2 cytokine production. This has prompted a shift in terminology from “TH2-high” to “type 2-high” asthma [7]. Roughly half of asthma patients exhibit this eosinophil-rich, type 2-high profile [8]. The remainder—classified as “type 2-low” asthma—lack eosinophilia and often display airway neutrophilia, obesity-related systemic inflammation, or minimal immune activation [9].

Asthma management underwent a paradigm shift in the early 2000s with the advent of the first biologic therapies (omalizumab). Before this, oral corticosteroids represented the ultimate treatment option for severe cases. Chronic use of systemic steroids, however, carries a substantial risk of serious side effects, ranging from adrenal suppression and Cushingoid features to glaucoma, cataract formation, steroid-induced diabetes, and bone demineralization [10]. Moreover, oral steroids fail to halt the structural changes in the airway driven by IL-33, a key cytokine in airway remodeling [11].

Even with strict adherence to conventional treatments, about 15 percent of asthma sufferers continue to experience poor disease control. For these individuals, both GINA and the National Heart, Lung, and Blood Institute recommend escalating to biologic agents [11]. Such therapies are indicated when patients’ symptoms remain uncontrolled or deteriorate despite optimized use of inhaled β_2_-agonists, anticholinergics, and corticosteroids—whether administered singly or in combination [12]. Clinical trials and real-world studies suggest that initiating a biologic is particularly beneficial in patients requiring two or more courses of oral steroids annually to manage exacerbations [13].

Prior to starting a biologic, confirming the asthma diagnosis and assessing biomarkers, such as peripheral eosinophil counts, total IgE, and fractional exhaled nitric oxide (FeNO), is essential. These measures help predict which patients are most likely to derive benefit from targeted biologic interventions [14]. Data from the International Severe Asthma Registry indicate that over 60 percent of enrolled patients meet eligibility criteria for biologics and experience high exacerbation rates; upon starting biologic therapy, their exacerbation frequency drops markedly [15]. Conversely, discontinuation of biologic treatment is associated with worsened outcomes and increased emergency healthcare utilization compared with those who maintain therapy [16]. As the role of biologics in severe asthma continues to broaden, further studies are required to define optimal treatment algorithms and long-term management strategies [17].

## 2. Asthma

### 2.1. Pro-Inflammatory and Anti-Inflammatory Aspects of Asthma

Under the influence of chemotactic signals released in the lungs, a variety of inflammatory cells migrate from the bloodstream into the airway tissue, where they contribute to asthma pathogenesis. In addition to these recruited leukocytes, resident structural cells, such as the airway epithelium, fibroblasts, and smooth muscle cells, serve as important sources of inflammatory mediators, actively sustaining the inflammatory milieu. In asthmatic patients, both arms of the immune system are engaged: innate effectors (mast cells, dendritic cells, eosinophils, neutrophils, basophils, innate lymphoid cells, monocytes, and macrophages) and adaptive lymphocytes (T and B cells) form the cellular landscape of airway inflammation [18].

Central to the eosinophilic inflammation seen in many asthma phenotypes are TH2 lymphocytes, which secrete IL-4, IL-5, and IL-13 in large amounts [19]. IL-4 drives allergic sensitization and class switching to IgE, IL-5 sustains eosinophil survival, and IL-13 orchestrates multiple pathological changes, mucus hypersecretion, goblet cell transformation, bronchial hyperresponsiveness, and airway remodeling [20]. By contrast, TH1 cells produce IL-2, IFN-γ, and TNF-α, which can counterbalance TH2-driven processes and thus may exert protective effects against eosinophil-dominant inflammation [21]. Supporting this, administration of IL-12 (a cytokine that promotes TH1 differentiation) in murine models reduces antigen-induced airway hyperresponsiveness and inflammation via IFN-γ-mediated pathways [22,23]. Yet, in some severe, corticosteroid-resistant asthma cases, an abundance of IFN-γ-producing TH1 cells has been linked to more intractable disease [24,25].

**Figure 1:**
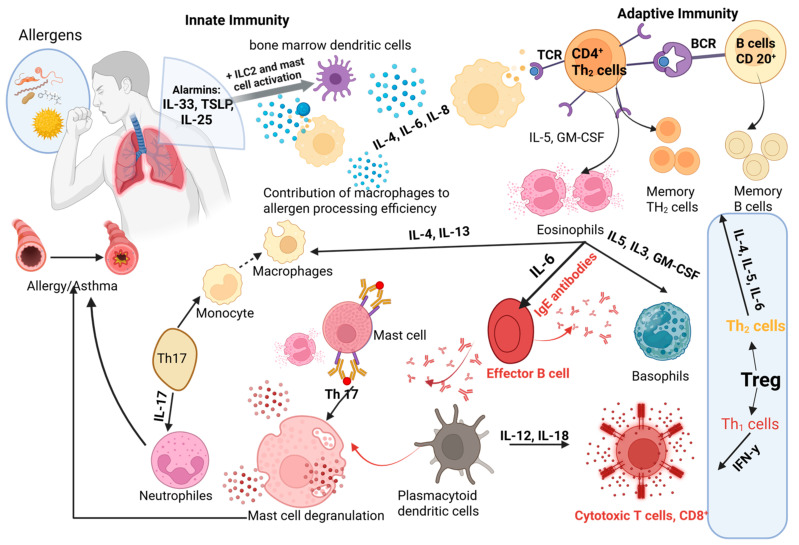
Immune cell differentiation and IgE-mediated responses in allergic asthma pathogenesis; Treg—regulatory T cells; Th1 cells—Type 1 helper T cells; IL-4—interleukin 4; IL-5—interleukin 5; IL-6—interleukin 6; IL-13—interleukin 13; IFN-γ —interferon gamma; GM-CSF—granulocyte-macrophage colony-stimulating factor. https://app.biorender.com/illustrations/6846a3f3ff2e9e10c41e8f5d (accessed on 9 June 2025).

Beyond TH2 and TH1 subsets, TH17 cells and their hallmark cytokine IL-17A have emerged as key players in neutrophil-predominant severe asthma phenotypes [26]. IL-17 and IL-22 enhance neutrophil recruitment to the airways [27], while neutrophil extracellular traps and cytoplasts further skew the immune response toward a TH17-driven, neutrophilic pattern [28]. However, IL-17’s role is dualistic: in some stages it confers protection, whereas in others it exacerbates airway inflammation [29,30]. In chronic models of asthma, IL-17A promotes fibroblast proliferation [31], undermines regulatory T cell-mediated suppression [32], and directly induces contraction of bronchial smooth muscle cells [33] (Figure 1).

The identification of TH9 cells, a CD4^+^ subset that secretes IL-9, has added another layer of complexity. Differentiation into TH9 is driven by IL-4 and TGF-β, which enable transcription factors PU.1 and IRF4 to activate the Il9 gene [34,35]. IL-25 (also known as IL-17E) further amplifies IL-9 production by TH9 cells [36]. Functionally, IL-9 promotes IgE synthesis and eosinophilic inflammation [37], and in experimental allergic models, mast cell accumulation and protease expression are IL-9 dependent [38,39]. Deletion of critical regulatory elements in the Il9 locus, which impairs TH9 maturation, markedly reduces allergic lung inflammation in mice [40,41]. Moreover, human trials of the anti-IL-9 monoclonal antibody MEDI-528 have shown promising efficacy in asthma patients [42].

Despite asthma’s well-recognized heterogeneity, a skewed immune balance underpins its development. It is the shifting interplay among T cell subsets and macrophage phenotypes, from disease initiation through progression, that shapes the overall inflammatory network. Rather than a simple overabundance of one cell type, it is the disruption of “pro-versus anti-inflammatory” regulatory circuits that drives pathology [43]. While the classic TH2-dominant paradigm remains fundamental, current evidence reveals a far more nuanced in vivo landscape, marked by uneven subpopulation dynamics and context-dependent cytokine expression.

### 2.2. Allergies and Asthma

The hallmark of allergy is an overzealous immune response to ordinarily harmless substances, pollen, house dust mites, pet dander or saliva, particular foods, and insect venoms among them. Upon exposure, sensitized individuals release mediators such as histamine, precipitating classic allergic manifestations: sneezing, itching, rhinorrhea or nasal congestion, tearing, and cutaneous eruptions. While most reactions are mild, severe anaphylaxis can occur [44].

Asthma and allergy frequently coexist, with allergens often acting as precipitating factors for bronchial hyperreactivity. In allergic asthma, inhaled triggers amplify airway inflammation, edema, and mucus secretion, further constricting the airways and provoking or intensifying wheezing and breathlessness.

Effective control of allergic asthma hinges on rigorous allergy management. It was demonstrated, as in many locales, that a high proportion of asthma sufferers exhibit atopic sensitization. Multiple epidemiological surveys have aimed to quantify asthma and its allergic subtype. For instance, Al Ghobain et al. (2018) used the ECRHS questionnaire in 2405 participants, finding an 18.2 percent rate of wheezing in the past year (absent upper respiratory infection), with no significant sex differences (*p* = 0.107) [45]. Physician-diagnosed asthma was reported by 11.3 percent (*p* = 0.239 between genders), and 10.6 percent were on asthma medications. Asthma prevalence did not vary significantly by residence (*p* = 0.07), education level (*p* = 0.11), or smoking status (*p* = 0.06), but showed a strong association with nasal allergy (*p* < 0.001). Long recognized as linked entities, asthma and allergic rhinitis share underlying immune mechanisms. Emerging evidence suggests that intense lower-airway inflammation can provoke or exacerbate upper-airway allergic responses, underscoring the continuum of airway disease in atopic individuals [46].

### 2.3. Cytokines in Asthma

T helper lymphocytes play a pivotal role in orchestrating immune reactions. In asthma, the interplay of cytokines, immune cell populations, and their signaling cascades forms a highly dynamic, intricate network. Cytokines, small protein mediators released by various immune cells, are critical for regulating inflammation, shaping immune responses, and facilitating cell-to-cell communication. Table 1 summarizes the key cytokines implicated in asthma and describes their respective contributions to the disease’s inflammatory and immunological processes.

In addition to classical Th2 cytokines such as IL-4, IL-5, and IL-13, the epithelial-derived alarmin thymic stromal lymphopoietin (TSLP) has emerged as a critical upstream mediator of airway inflammation in asthma, and is now recognized as a major therapeutic target, most notably for the monoclonal antibody tezepelumab.

### 2.4. Exacerbations in Asthma

Asthma flare-ups significantly contribute to patient morbidity, elevate healthcare expenditures, and, for some individuals, accelerate the decline in pulmonary function [60]. Although exacerbation rates can be lowered through optimized therapies, complete prevention is not always achievable [61]. Since acute attacks may occur despite adherence to standard regimens, early recognition of high-risk patients and implementation of personalized action plans are vital for improving overall control and quality of life.

Episodes leading to emergency department visits or hospital admissions are not only costly but also predict a higher likelihood of future exacerbations, regardless of baseline asthma severity, demographic factors, or previous control levels [62]. This underscores the ongoing necessity for more effective preventive and therapeutic strategies.

The most frequent precipitants of these attacks are viral respiratory pathogens, especially human rhinovirus subtypes A and C [63]. In school-aged children, and similarly in adults, hospital admissions for asthma spike in autumn (September–December) and again in spring, mirroring seasonal peaks in rhinovirus circulation [64,65].

Beyond rhinoviruses, other viruses play roles in acute worsening. During the 2009 H1N1 influenza A pandemic, asthma patients represented a disproportionate share of ICU admissions and fatalities [66,67]. Respiratory syncytial virus, well known for causing wheezing in infants, can also trigger exacerbations in older adults, particularly those over 65 [68]. Although coronaviruses, metapneumoviruses, parainfluenza viruses, adenoviruses, and bocaviruses are detected less commonly in asthma attacks, their presence has been confirmed [69].

Bacterial infections may impair mucociliary clearance and heighten mucus production, potentially sustaining chronic airway inflammation [70]. While direct links between bacteria and sudden asthma flares remain limited, viral infections can weaken macrophage-mediated antibacterial defenses and alter the airway microbiome, which could predispose to secondary bacterial involvement [71].

Atopic sensitization further compromises innate antiviral responses, rendering individuals more susceptible to virus-induced wheezing; allergen-driven inflammation also amplifies airway reactivity to rhinoviruses. In addition, sensitization to molds such as Alternaria alternata, which correlates with five-fold higher odds of asthma, increased bronchial hyperresponsiveness, wheezing, and inhaler use, aligns with seasonal exacerbation trends tied to airborne spore counts [72].

Environmental irritants likewise act as triggers. Tobacco smoke exposure is linked to persistent wheezing, more severe asthma, and a higher rate of ED visits and hospitalizations [73]. Pollutants, including particulate matter, ozone, nitrogen dioxide, sulfur dioxide, and diesel exhaust, intensify airway inflammation and hyperreactivity and can synergize with viral infections to provoke attacks [74].

## 3. Targeted Immune Modulation in Severe Asthma

### 3.1. Conventional Biomarkers in Clinical Practice

Asthma management increasingly relies on measurable indicators, such as eosinophil and neutrophil counts, total IgE, fractional exhaled nitric oxide (FeNO), leukotriene levels, and others, that bridge underlying biology with clinical presentation. Although no single marker is without limitations, these tests are invaluable for tailoring the use of targeted biologic treatments.

#### 3.1.1. Eosinophils

Peripheral blood and sputum eosinophil counts correlate with disease activity in both atopic and non-atopic asthma. Elevated eosinophils signal greater severity, and serial blood measurements are routinely used to monitor the response to anti-IL-5 therapies [75]. To date, blood eosinophilia remains one of the most reliable biomarkers for classifying asthma subtypes and gauging treatment effectiveness.

#### 3.1.2. Neutrophils

High airway neutrophil levels often characterize severe, non-type 2 asthma phenotypes driven by TH17 pathways [76]. While sputum analysis can reveal pronounced neutrophilia, the relationship between airway and blood neutrophil counts is inconsistent in some cohorts [77], underscoring the need for complementary assessments.

#### 3.1.3. Total IgE

IgE production is promoted by TH2 lymphocyte–B cell interactions, leading to mast cell and basophil activation [78]. Although serum IgE does not reliably track disease severity or therapeutic response, anti-IgE monoclonal antibodies are approved for patients with persistent atopy, particularly children with allergic asthma and adults who also suffer from allergic rhinitis, chronic sinusitis, nasal polyposis, or chronic urticaria [79].

#### 3.1.4. Fractional Exhaled Nitric Oxide (FeNO)

Nitric oxide synthesis in the airway epithelium is upregulated by IL-4 and IL-13 signaling via IL-4Rα [76]. FeNO measurement offers a noninvasive readout of eosinophilic inflammation and type 2-driven asthma; it can be performed at home to guide therapy adjustments [80]. Together, FeNO and blood eosinophil levels form the cornerstone of biomarker-guided management in severe asthma.

#### 3.1.5. Leukotrienes

Leukotrienes are synthesized through the 5-lipoxygenase (5-LOX) enzymatic route. These cysteinyl leukotrienes contribute to bronchoconstriction and drive inflammatory processes. In asthmatic airways, they provoke smooth muscle tightening, increase vascular leak and mucus output, and recruit plus activate multiple inflammatory cell types [81].

### 3.2. Exacerbation with Biologics

Biologic agents have proven their worth in cutting the frequency of severe asthma exacerbations [82]. In the MEX trial, researchers characterized the nature of breakthrough exacerbations in patients with severe eosinophilic asthma receiving mepolizumab. Despite profound reductions in circulating eosinophils, about half of these residual attacks were still driven by eosinophilic inflammation, while the other half showed a non-eosinophilic profile [83]. Importantly, FeNO measurements taken during an exacerbation reliably distinguished high-eosinophil from low-eosinophil events. When FeNO was ≤20 ppb, the negative predictive value for an eosinophilic flare was 100 percent (95% CI 0.8–1.0), suggesting that systemic steroids might be avoidable in this setting. Conversely, FeNO ≥ 50 ppb corresponded to a positive predictive value of 76.9 percent (95% CI 0.6–0.9) for eosinophilic attacks, indicating that oral corticosteroids were likely warranted [84].

A separate prospective investigation by Poznanski et al. evaluated why some patients on benralizumab continued to experience exacerbations or failed to halve their prednisone dose, a definition of suboptimal response, over a median follow-up of 14 months [85]. Among 74 treated individuals (23 of whom underwent NK cell quantification), 27 percent met the suboptimal response criteria. These patients’ sputum was neutrophil-rich (mean 19.5 ± 37 × 10^6^ cells/g vs. 4.5 ± 6 × 10^6^ cells/g in responders; *p* = 0.01) and frequently linked to viral or bacterial infections (e.g., *H. influenzae*, *M. catarrhalis*, *S. pneumoniae*, *S. aureus*) [84]. They also had experienced more infections in the prior year (median 0.5 vs. 0) and exhibited lower circulating NK cell counts alongside elevated NKT cell numbers, cells known to drive neutrophil recruitment and activation.

Together, these findings underscore that even under targeted T2-inflammation blockade, persistent exacerbations remain heterogeneous. Profiling each flare, distinguishing eosinophilic from neutrophilic phenotypes, emerges as a critical step toward precision interventions and, ultimately, achieving remission in severe asthma [86].

### 3.3. Anti-IgE: Omalizumab

#### 3.3.1. Mechanism of Action and Efficacy

**Figure 2:**
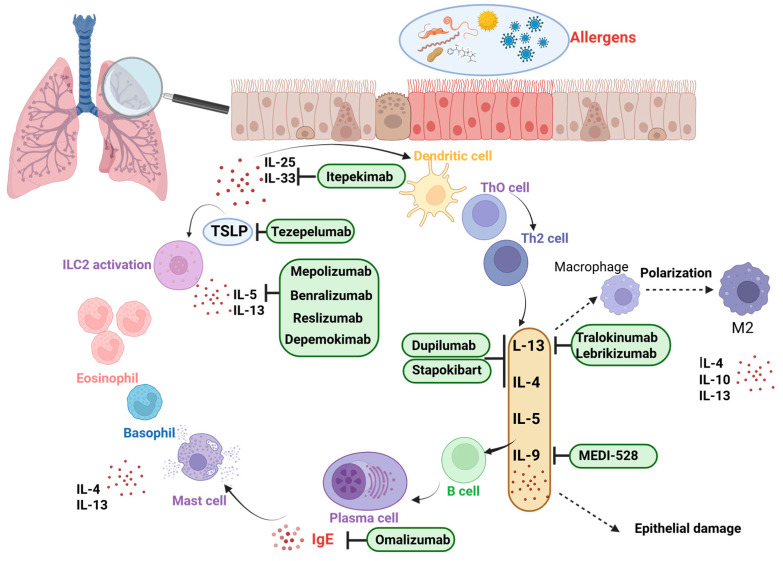
Molecular targets of biologic therapies in type 2-high asthma: cytokine pathways and immune cell interactions. TSLP—thymic stromal lymphopoietin; ILC2—group 2 innate lymphoid cells; Th2 cell—T helper 2 cell; IgE—immunoglobulin E; https://app.biorender.com/illustrations/68503e3f1998ef5af7e0af7a (accessed on 9 June 2025).

Omalizumab is a recombinant humanized monoclonal antibody targeting immunoglobulin E (IgE), and it was the first biologic to receive approval for asthma treatment in both the U.S. and Europe. Since approximately 70% of asthma cases are linked to allergic mechanisms, IgE plays a pivotal role in the inflammatory cascade underlying these conditions [87,88]. Produced by B lymphocytes in response to allergen exposure, IgE binds to high-affinity receptors (FcεRI) located on mast cells and basophils, promoting the release of inflammatory mediators. Omalizumab interrupts this process by blocking IgE from binding to its receptor, thereby attenuating downstream allergic signaling [89,90]. Additionally, it reduces the expression of FcεRI receptors on effector cells, further dampening the immune response [90] (Figure 2).

Interestingly, beyond these established pathways, clinical research has noted a decrease in asthma exacerbations during viral seasons among patients on omalizumab. This effect has been associated with increased production of interferon-alpha (IFN-α) in response to rhinovirus, suggesting the antibody may also enhance antiviral immunity [91].

Omalizumab has been utilized in clinical practice for over 15 years, demonstrating consistent efficacy in allergic asthma management. A comprehensive Cochrane meta-analysis conducted in 2014 analyzed 25 randomized controlled trials and confirmed that omalizumab significantly reduced asthma exacerbations (by roughly 25%), decreased hospital admissions, and enabled reductions in inhaled corticosteroid (ICS) usage [92,93,94,95,96]. While some studies have noted slight improvements in pulmonary function, others did not observe significant changes [97]. Data supporting reductions in oral corticosteroid (OCS) use remain inconclusive.

Initial trials mainly included patients with moderate allergic asthma; however, more recent studies extended to severe cases, showing comparable effectiveness [98]. Observational studies from real-world settings have similarly reported fewer exacerbations and hospital visits among patients treated with omalizumab [99,100].

Ongoing investigations aim to identify patient profiles that respond best to this therapy. Retrospective assessments indicate that individuals with elevated eosinophil counts and exhaled nitric oxide (FeNO) levels experience more substantial reductions in exacerbation rates [101]. This may be attributable to their initially higher inflammation burden, allowing for greater visible improvement with treatment. Nonetheless, patients with low type 2 (T2) biomarker levels can also derive benefit from omalizumab. A recent pragmatic trial supported its effectiveness across both high and low T2 biomarker groups (absolute eosinophil count < 300 vs. ≥300 cells/μL and FeNO < 25 vs. ≥25 ppb) [100].

Further, the antibody has shown efficacy in patients with IgE levels outside the U.S.-approved range (30–700 IU/mL), including those above 700 IU/mL [100]. Notably, a pilot study found that omalizumab could downregulate FcεRI expression even in nonatopic asthma patients, opening the door for broader therapeutic applications [102].

#### 3.3.2. Indications, Administration, and Safety

In the U.S., omalizumab is authorized for patients aged 6 years and older who have moderate to severe persistent asthma, uncontrolled on ICS therapy, and show evidence of allergic sensitization. Eligibility includes total serum IgE levels ranging from 30 to 1300 IU/mL for children aged 6–11 and 30 to 700 IU/mL for those 12 and older (in the EU, the range extends to 1500 IU/mL).

Administration involves subcutaneous injections every 2 to 4 weeks, with dosing based on patient weight and baseline IgE levels. Routine monitoring of IgE during treatment is not advised. A trial period of 3–6 months is recommended to assess treatment response. If beneficial, long-term continuation is supported by findings from the XPORT study (Xolair Persistency of Response after Long-Term Therapy) [103]. Omalizumab is generally safe, with an anaphylaxis incidence estimated between 0.1% and 0.2% [104]. Due to this rare but serious risk, the FDA mandates a black box warning, and initial administrations should occur in clinical settings equipped to manage allergic emergencies. Patients are advised to remain under observation for 2 h following their first three injections, then for 30 min after subsequent doses (Table 2).

### 3.4. Anti-Interleukin-5 Monoclonal Antibodies

Targeting eosinophils in severe asthma requires understanding both circulating and airway-resident eosinophil dynamics. While peripheral eosinophil counts (AECs) generally mirror sputum eosinophil levels in patients on low-to-moderate inhaled corticosteroids [108], this correlation breaks down in those maintained on systemic steroids [109]. Sustained AECs above 400 cells/µL usually indicate sputum eosinophilia, but normal AECs do not rule it out. Such mismatches hint at local eosinophil-driven inflammation within the airway wall, mechanisms that anti-IL-5 therapy may not fully suppress and which can perpetuate symptoms [110,111,112] (Figure 2).

Weight-based IV reslizumab demonstrated more uniform suppression, both sputum eosinophils and eosinophil peroxidase levels declined, and asthma control improved [113]. However, without head-to-head trials comparing mepolizumab, reslizumab, and benralizumab under identical enrollment criteria, definitive conclusions about optimal dosing and agent selection remain elusive.

Intriguingly, patients on low-dose mepolizumab sometimes show increased free IL-5 and IgG–IL-5 immune complexes in sputum, despite the presence of drug–cytokine aggregates. Such complexes can paradoxically enhance the bioactivity of IL-5 in vivo, potentially worsening inflammation [114]. These observations, concurrent rises in ILC2 counts, free and Ig-bound IL-5 in those who deteriorate on low-dose anti-IL-5 therapy, underscore the need to tailor both dosing and agent choice to each patient’s eosinophil biology [114]. In one study, a single 750 mg IV dose of mepolizumab in patients with persistent sputum eosinophilia markedly lowered both blood and sputum eosinophils, enabled an 87 percent reduction in oral steroid requirements, and improved asthma control [115]. By contrast, the SIRIUS trial showed only modest steroid-sparing effects when mepolizumab was given as 100 mg SC injections [116]. A small series of ten patients on the 100 mg dose experienced significant falls in AEC but had persisting or even increased sputum eosinophils that correlated with clinical exacerbations [111]. This suggests low-dose SC mepolizumab may not fully inhibit airway eosinophilopoiesis, likely driven by local IL-5–producing ILC2s and eosinophil progenitors [111,112] (Table 3).

#### 3.4.1. Mepolizumab

Eosinophil activation within the bronchial walls contributes to airway constriction, excessive mucus production, and structural changes over time. Consequently, therapies targeting eosinophils have been central to asthma care. Among the many cytokines and surface molecules that govern eosinophil survival, growth, and activation, interleukin-5 (IL-5) and its receptor have been studied most intensively (Figure 2).

Mepolizumab (brand name Nucala) became the inaugural anti-IL-5 monoclonal antibody approved for adjunctive, long-term treatment of severe eosinophilic asthma. By blocking IL-5 from engaging its receptor (IL-5Rα) on eosinophils, mepolizumab disrupts the maturation of these cells in the bone marrow and leads to reduced deposition of extracellular matrix components in the reticular basement membrane of the airway mucosa. Mepolizumab has a favorable safety profile, with the most common adverse effects being headache, injection site reactions, and back pain. Hypersensitivity reactions are rare. Long-term safety data have not revealed new safety signals [121] (Table 4).

In addition to severe eosinophilic asthma, mepolizumab is also approved for the treatment of eosinophilic granulomatosis with polyangiitis (EGPA), further broadening its clinical utility in patients with overlapping comorbidities [122].

**Table 4:** Key studies supporting the clinical use of mepolizumab in severe asthma.

Study/Trial Name	Study Type	Key Findings	Reference
General IL-5 Research	Mechanistic/Preclinical	IL-5 is critical for eosinophil activation, recruitment, and survival.	[123]
DREAM Study (Phase 2)	Randomized Controlled Trial	Identified elevated eosinophils and prior exacerbations as predictors for better response to Mepolizumab.	[124]
SIRIUS Trial (Phase 3)	Randomized Controlled Trial	Showed reduction in oral corticosteroid (OCS) use with Mepolizumab therapy.	[125]
MUSCA Study (Phase 3)	Randomized Controlled Trial	Demonstrated improved quality of life and safety profile comparable to placebo.	[126]
MENSA Trial (Phase 3)	Randomized Controlled Trial	Reported a 53% reduction in asthma exacerbations with Mepolizumab vs. placebo in patients on high-dose ICS + LABA.	[124]
Clinical Use Overview	Real-world/Approved Indication	Approved for patients ≥ 6 years old with eosinophils ≥ 150 cells/μL; administered as 100 mg SC every 4 weeks.	[124,125,126,127]
Comparative Use Study	Observational/Clinical Practice	Recommended for patients with poor response to Omalizumab; effective in both allergic and non-allergic asthma.	[128,129]
Off-label Application	Regulatory/Clinical Summary	Currently the only approved anti-IL-5 agent for eosinophilic granuloma and vasculitis.	[130]

#### 3.4.2. Reslizumab

Reslizumab (brand name Cinqair) gained FDA approval in 2016 as an add-on therapy for severe eosinophilic asthma. Its action hinges on binding free interleukin-5, thereby preventing this cytokine from engaging its receptor on eosinophils (Figure 2). Since IL-5 is a key driver of eosinophil maturation, recruitment, activation, and longevity, sequestering it disrupts these processes and leads to a reduction in eosinophil numbers and activity. The most frequently reported adverse effects are oropharyngeal pain, increased creatine phosphokinase, and myalgia. Anaphylaxis has been reported in rare cases. A comprehensive evaluation of the long-term safety profile is still ongoing [131] (Table 5).

#### 3.4.3. Benralizumab

Benralizumab is a humanized monoclonal antibody that binds selectively to the α-chain of the interleukin-5 receptor (IL-5Rα), blocking IL-5 signaling on eosinophils, basophils, and ILC2s (Figure 2). By engaging FcγRIIIa on natural killer cells, it induces antibody-dependent cell-mediated cytotoxicity, leading to targeted eosinophil apoptosis [137]. The drug is approved for patients aged 12 years and older with moderate to severe asthma and a baseline peripheral blood eosinophil count of at least 150 cells/µL.

Benralizumab is indicated for individuals aged 12 years and above whose asthma remains uncontrolled and who have an absolute eosinophil count exceeding 300 cells/µL [138]. The recommended regimen begins with a 30 mg subcutaneous injection once every four weeks for the initial three doses, an induction phase aimed at depleting eosinophils in the tissues, followed by maintenance injections every eight weeks. A minimum treatment period of four months is advised to evaluate therapeutic effectiveness. Overall, benralizumab is well tolerated, although rare hypersensitivity events such as anaphylaxis, angioedema, and urticaria have been reported (Table 6).

More recently, benralizumab has also demonstrated efficacy in eosinophilic granulomatosis with polyangiitis (EGPA), providing an alternative therapeutic option for patients with this comorbidity [139].

**Table 6:** Summary of key clinical evidence supporting benralizumab in severe eosinophilic asthma.

Study/Trial Name	Study Type	Key Findings	Reference
General Efficacy Overview	Clinical Recommendation	Approved for patients aged ≥ 12 with eosinophils > 300 cells/μL unresponsive to GINA Step 4–5 therapies.	[140]
SIROCCO Trial (Phase 3)	RCT, Placebo-Controlled (*n* = 1204)	Achieved 51% reduction in annual asthma exacerbations and improved FEV_1_ in patients with high eosinophil counts.	[141]
CALIMA Trial (Phase 3)	RCT	Showed reduced exacerbations and enhanced lung function after 56 weeks of treatment.	[142]

### 3.5. Dupilumab

Dupilumab exerts its effects by binding to the α-subunit of the interleukin-4 receptor (IL-4Rα), thereby simultaneously interrupting both IL-4 and IL-13 signaling pathways. The type 1 IL-4 receptor, composed of IL-4Rα paired with the common γ-chain, is expressed on B and T lymphocytes, monocytes, eosinophils, and fibroblasts (Figure 2). The type 2 receptor, a heterodimer of IL-4Rα and IL-13Rα1, is found on monocytes, fibroblasts, eosinophils, activated B cells, epithelial and goblet cells, and airway smooth muscle. IL-4 can engage both receptor types, whereas IL-13 signals solely through the type 2 complex. By targeting the shared IL-4Rα component, dupilumab blocks downstream cascades initiated by both cytokines [143]. IL-4 is crucial for TH2 differentiation and expansion, B-cell proliferation, class-switch recombination to IgE, and eosinophil migration. IL-13 overlaps in function but additionally drives goblet cell hyperplasia, mucus hypersecretion, and smooth muscle contraction and hypertrophy. Both cytokines also induce epithelial and endothelial cells to secrete chemokines such as eotaxin-3, which guides eosinophils to inflamed tissues. IL-4 further upregulates adhesion molecules on the endothelium, facilitating eosinophil extravasation under the direction of chemokines like TARC [144,145,146]. Because IL-4 and IL-13 share many activities, inhibiting only one of these cytokines yields incomplete suppression of type 2 inflammation. Dupilumab’s dual blockade of IL-4 and IL-13 signaling via IL-4Rα is therefore necessary to fully interrupt key pathogenic processes—TH2-driven antigen presentation, eosinophil recruitment to the lungs, and the production of inflammatory mediators and chemokines. By targeting this central IL-4/IL-13 axis, dupilumab addresses fundamental mechanisms common to a range of type 2 inflammatory disorders, mitigating epithelial hyperplasia, basement membrane thickening, barrier disruption, and tissue infiltration that underlie clinical symptoms. The safety profile of dupilumab is well established; most adverse events are mild, including injection site reactions and transient eosinophilia. Conjunctivitis is also reported more frequently than with a placebo [147] (Table 7). Recent evidence suggests that dupilumab may be effective even in patients who have not achieved adequate disease control with anti-IL-5 or anti-IL-5R therapies. In a recent study, patients with severe eosinophilic asthma who experienced a suboptimal response to anti-IL-5/5R biologics demonstrated clinically meaningful improvements following a switch to anti-IL-4R therapy with dupilumab [148].

### 3.6. Tezepelumab

Tezepelumab (TZP) is a fully human IgG2κ monoclonal antibody designed to occupy the thymic stromal lymphopoietin (TSLP) binding site required for heterodimeric TSLP receptor (TSLPR) engagement, thereby blocking TSLP–TSLPR signaling (Figure 2) [156]. Both TSLPR and interleukin-7 receptor (IL-7R) co-localize on eosinophil surfaces, rendering these cells responsive to TSLP’s survival and activation cues, which include release of cytotoxic granule proteins and proinflammatory chemokines [157,158]. Signaling downstream of TSLPR normally proceeds via MAP kinase and NF-κB pathways, but TZP’s antagonism interrupts this cascade, resulting in broad suppression of cytokine production and inflammatory cell activation [158]. Clinical studies have demonstrated that TZP significantly lowers tissue eosinophil counts, blood eosinophil counts (BECs), total IgE, IL-5, IL-13, and fractional exhaled nitric oxide (FeNO), while also promoting restoration of epithelial barrier integrity [159]. Furthermore, recent real-world evidence suggests that higher baseline T2 biomarker levels are associated with a greater likelihood of clinical remission with tezepelumab in patients with severe uncontrolled asthma [160]. In 2018, the U.S. FDA granted tezepelumab breakthrough therapy status for patients with severe asthma lacking a high type 2 phenotype, who remain symptomatic despite treatment with inhaled corticosteroids plus long-acting β_2_-agonists (with or without oral corticosteroids) and additional controllers [161]. To date, TZP stands alone among biologics in offering targeted therapy for type 2–low asthma, though it may also be considered for patients exhibiting both atopy and eosinophilia. Tezepelumab is associated with a low rate of adverse events, most commonly nasopharyngitis and headache. No new safety concerns have been identified in ongoing extension studies, but safety profile evaluation is still ongoing.

In addition to asthma, tezepelumab has shown clinical benefit in patients with nasal polyposis, expanding its potential indications, particularly in those with overlapping upper and lower airway disease [162].

## 4. Perspectives

### 4.1. Depemokimab-Anti-IL5

Surveys and discrete choice experiments across various therapeutic fields, including severe asthma, consistently show that both patients and clinicians favor regimens requiring fewer injections [163]. Depemokimab, a next-generation anti-IL-5 monoclonal antibody, exhibits roughly 29-fold greater potency and about half the clearance rate of mepolizumab in single-dose pharmacokinetic and pharmacodynamic evaluations performed in cynomolgus monkeys (GSK data on file) (Figure 2). In an initial human trial involving individuals with mild to moderate asthma and baseline blood eosinophil counts ≥ 200 cells/μL, subcutaneous depemokimab (doses 2–300 mg) displayed a favorable safety profile, predictable, dose-proportional pharmacokinetics, and an extended terminal half-life of 38–53 days, substantially longer than the 16–22 days for mepolizumab and 15.5 days for benralizumab, supporting dosing intervals of up to six months [164,165]. That first-in-human study used subcutaneous injections prepared from vials by specialized laboratory and pharmacy teams [164]. In routine practice, most biologics for severe asthma are delivered subcutaneously, either in the patient’s home or a clinic, via a prefilled syringe, which can be incorporated into a safety syringe device (SSD) or an autoinjector (AI) [166]. With an SSD, the user inserts the needle into the skin, depresses the plunger to administer the drug, and the needle automatically retracts into the housing afterward. In contrast, an AI conceals the needle entirely and deploys the medication automatically upon activation. Both delivery systems facilitate convenient therapy administration, though individual preferences among patients and healthcare providers may differ based on factors such as simplicity of use and perceived injection discomfort [167,168,169].

Existing biologic treatments for asthma require injections every four to eight weeks [170]. Due to its prolonged elimination half-life, depemokimab has the potential to extend this interval further. Currently, multiple Phase III studies are evaluating depemokimab in various eosinophil-driven conditions: asthma (NCT05243680, NCT04718389), chronic rhinosinusitis with nasal polyps (NCT05274750, NCT05281523), eosinophilic granulomatosis with polyangiitis (NCT05263934), and hypereosinophilic syndrome (NCT05334368). A separate Phase III trial, NIMBLE, is investigating the effects—both in terms of efficacy and safety—of switching patients from existing anti-IL-5 therapies (mepolizumab or benralizumab) to depemokimab, with topline data anticipated in late 2026. In addition, allowing patients to choose among several injection sites can be beneficial for personal comfort and practicality during self-administration, especially if certain areas become tender or hard to reach [167,171]. Pharmacokinetic analyses have shown that depemokimab’s absorption and disposition remain consistent whether administered into the upper arm, abdomen, or thigh, independent of the delivery device. Therefore, offering multiple, rotatable injection locations provides greater flexibility and autonomy in patient self-management.

### 4.2. Itepekimab

IL-33 functions as an alarmin released from airway epithelial and endothelial cells when they experience stress or injury caused by inhaled allergens, viral infections, tobacco smoke, or pollutants. Once secreted, IL-33 binds to the ST2 receptor on immune cells, recruiting the IL-1 receptor accessory protein (IL-1RAcP) and triggering downstream MAP kinase and NF-κB signaling. This activation cascade promotes the secretion of proinflammatory cytokines that initiate and amplify both innate and adaptive immune responses [172] (Figure 2). The specific roles of IL-33, together with other epithelial-derived alarmins such as IL-25 and thymic stromal lymphopoietin, in sustaining chronic airway inflammation in diseases like asthma and COPD, as well as the degree to which these alarmins overlap or compensate for one another, remain active areas of research [173].

Itepekimab (formerly REGN3500 and SAR440340) is a fully human IgG4P monoclonal antibody that targets IL-33. It was developed using VelocImmune^®^ technology, which employs a specially engineered mouse with a humanized immune system to generate high-affinity human antibodies [174,175]. The IgG4P format features a serine-to-proline mutation in the hinge region, promoting stable dimer formation [176]. In a preclinical house dust mite (HDM) mouse model of airway inflammation, itepekimab’s blockade of IL-33 significantly attenuated lung tissue injury and reduced inflammatory cell infiltration, supporting its potential to disrupt epithelial-driven inflammatory pathways in respiratory diseases [177]. Currently, the Phase III AERIFY-1 (NCT04701983) and AERIFY-2 (NCT04751487) trials are evaluating itepekimab’s safety and efficacy in COPD patients.

Pharmacokinetic analyses revealed that, after a single intravenous dose in healthy volunteers and following multiple doses in individuals with moderate asthma, itepekimab displayed linear, dose-proportional kinetics and a long elimination half-life, consistent with once-monthly or less frequent dosing schedules. Early single-dose cohorts (0.3 mg/kg) prompted extended follow-up for later cohorts to ensure thorough characterization of PK and pharmacodynamic (PD) endpoints [178]. No treatment-emergent anti-drug antibodies (ADAs) were detected, and itepekimab was generally well tolerated across all studies.

Baseline total IL-33 levels in serum were typically undetectable in both healthy subjects and those with asthma, mirroring findings in COPD populations with low systemic IL-33 concentrations [179]. Upon itepekimab administration, serum IL-33 levels rose, reflecting formation of a stable IL-33–itepekimab complex. Given the negligible free IL-33 at baseline, the increase in detectable total IL-33 post-treatment likely represents bound alarmin, serving as a pharmacodynamic marker of target engagement. Notably, the decline of total IL-33 concentrations occurred more slowly than itepekimab’s own half-life, suggesting that the clearance of the IL-33–itepekimab complex is protracted relative to the antibody alone. Dose-dependent elevations in total IL-33 were seen in the single-dose study; profiles for participants receiving 3 mg/kg versus 10 mg/kg intravenous doses overlapped, indicating saturation of IL-33 binding at higher doses [180].

In summary, itepekimab exhibits favorable tolerability, robust subcutaneous absorption, linear and dose-proportional pharmacokinetics, and no detectable ADA induction in both healthy volunteers and moderate asthma patients. The observed increases in total serum IL-33 confirm effective target engagement, and concomitant reductions in blood eosinophil counts demonstrate pharmacodynamic activity.

### 4.3. CM310-Stapokibart

Stapokibart is a humanized monoclonal antibody directed against IL-4Rα, engineered to inhibit both IL-4 and IL-13 signaling, in contrast to dupilumab, which is fully human (Figure 2). Its safety and efficacy have been evaluated in healthy volunteers and in patients with atopic dermatitis (AD) or chronic rhinosinusitis with nasal polyps (CRSwNP) during phase 1b/2a (NCT04893941), phase 2b (NCT04805411), and phase 2 (NCT04805398) trials. In a study targeting severe eosinophilic CRSwNP, stapokibart significantly decreased nasal polyp size and improved nasal congestion, both coprimary endpoints [181]. For moderate to severe AD, a phase III trial demonstrated that 66.9 percent of stapokibart-treated participants achieved EASI-75 and 44.2 percent reached an IGA score of 0/1 (≥2-point reduction), outperforming placebo rates of 25.8 percent and 16.1 percent, respectively [182]. Details regarding stapokibart’s underlying mechanism and preclinical data have not yet been publicly disclosed. Although stapokibart and dupilumab have not been directly compared in the same trial, separate studies suggest comparable efficacy in type 2 inflammatory conditions. In the CROWNS-1 phase II trial for severe CRSwNP, 79 percent (22/28) of patients receiving stapokibart experienced at least a 2-point reduction in Nasal Polyp Score by week 16 [181]. By comparison, dupilumab achieved this outcome in 46 percent of participants in the SINUS-24 trial (66/143) and 46 percent in SINUS-52 (136/295) [183]. For moderate to severe AD, 66.9 percent of stapokibart recipients met EASI-75 in its phase III study, versus 57.3 percent for dupilumab in a separate trial with a similar ethnic composition; placebo rates were 25.8 percent and 14.5 percent, respectively. Similarly, IGA 0/1 (≥2-point reduction) occurred in 44.2 percent of stapokibart-treated subjects compared to 26.8 percent with dupilumab, while placebo groups showed 16.1 percent and 4.8 percent, respectively [184]. Definitive head-to-head trials are needed to confirm these observations. In vitro assays reveal stapokibart’s ability to block IL-4 and IL-13/IL-13Rα1 binding to IL-4Rα, matching or slightly surpassing dupilumab’s neutralizing potency. In vivo pharmacology studies demonstrate that stapokibart’s IL-4/IL-13 blockade effectively protects rats in various type 2 allergy models. Toxicology evaluations further indicate a favorable safety margin, with a no-observed-adverse-effect level (NOAEL) of 150 mg/kg when dosed weekly for 26 weeks.

### 4.4. Tralokinumab

Tralokinumab underwent evaluation in three Phase II studies involving asthma patients (NCT00640016, NCT00873860 [185], and NCT01402986 [186]), although one trial was terminated early due to enrollment difficulties. In the two trials that completed enrollment (NCT00873860 and NCT01402986), the primary endpoints were not reached when assessing all enrolled participants. Nonetheless, both studies demonstrated that inhibiting IL-13 with tralokinumab led to improvements in pulmonary function among individuals with moderate to severe asthma that was not adequately controlled on medium- or high-dose ICS/LABA. Post hoc analyses from these trials revealed that patients exhibiting biomarkers indicative of IL-13 activity experienced greater clinical benefit [185,186]. In the Phase IIa trial (NCT00873860), a subgroup of moderate to severe uncontrolled asthmatics who had elevated sputum IL-13 at baseline showed trends toward meaningful enhancements in Asthma Control Questionnaire (ACQ-6) scores and FEV_1_ when compared with placebo [185] (Figure 2). Recognizing that sputum collection is impractical in larger studies, the Phase IIb trial (NCT01402986) instead used serum periostin and DPP-4 levels as surrogate markers for IL-13 signaling [186]. Post hoc findings in this cohort, specifically participants with severe uncontrolled asthma, baseline FEV_1_ reversibility ≥ 12%, no regular OCS use at entry, and elevated DPP-4 or periostin, showed that 300 mg of tralokinumab administered subcutaneously every two weeks yielded improvements in lung function, exacerbation frequency, symptom control, and quality of life [186]. Across both Phase II trials, no new safety concerns emerged: in the Phase IIa study, no serious adverse events (AEs) were attributed to tralokinumab, and in Phase IIb, treatment-emergent AEs occurred at similar rates in both tralokinumab and placebo arms [185].

A combined pharmacokinetic–pharmacodynamic (PK–PD) model applied to these Phase II data predicted that a 300 mg subcutaneous dose every two weeks would achieve near-maximal FEV_1_ increases, leading to its selection for the Phase III program [187]. The ATMOSPHERE trials were designed to identify whether a subset of patients would exhibit an enhanced response to tralokinumab, potentially enabling more targeted management of IL-13-driven asthma. Previous attempts with other IL-13-targeted agents have produced mixed results: lebrikizumab, for instance, failed to show consistent benefit in both all-comers and serum periostin- or eosinophil-high subgroups during Phase III trials [188], whereas dupilumab, targeting both IL-4 and IL-13, demonstrated efficacy in a broad severe asthma population as well as in those with elevated blood eosinophils in a Phase IIb trial [189]. The experience with tralokinumab mirrors that of lebrikizumab, as no clearly defined patient subset has yet been identified that consistently achieves a robust, clinically meaningful response (Table 8).

### 4.5. Lebrikizumab

Lebrikizumab is an IgG4 monoclonal antibody that binds IL-13 with very high affinity and a slow dissociation rate, selectively blocking IL-13 signaling via the IL-13Rα1/IL-4Rα receptor complex and thus potently inhibiting downstream IL-13-mediated effects. Because its activity is confined to IL-13 neutralization, lebrikizumab was hypothesized to benefit patients exhibiting a type 2 inflammatory signature (Figure 2). To evaluate this, trials were conducted in individuals with moderate to severe asthma that remained uncontrolled despite standard therapies. However, Phase III studies did not consistently demonstrate reductions in exacerbation rates across the broad asthmatic population [193,194], calling into question whether isolated IL-13 blockade, without concurrent IL-4 inhibition, would suffice in difficult-to-treat cases. In the ACOUSTICS trial, adolescents received lebrikizumab over a 52-week, placebo-controlled period. Those treated with 125 mg (*n* = 116) showed a greater decrease in annualized exacerbation rate (AER) compared to placebo than did the 37.5 mg cohort (*n* = 113). Specifically, the adjusted rate ratios (RRs) versus placebo were 0.49 (95% CI, 0.28–0.83) for 125 mg and 0.60 (95% CI, 0.35–1.03) for 37.5 mg, corresponding to 51% and 40% reductions in AER, respectively. When focusing on participants with baseline blood eosinophils ≥ 300 cells/μL and at least one exacerbation in the prior year, the AER declined even further: RR = 0.41 (95% CI, 0.19–0.88) for 125 mg and RR = 0.36 (95% CI, 0.15–0.87) for 37.5 mg, representing reductions of 59% and 64% compared to placebo [193,194].

A secondary endpoint in the pooled LAVOLTA trials assessed lung function via absolute change from baseline in pre-bronchodilator FEV_1_. Post hoc analyses revealed that both 125 mg and 37.5 mg doses of lebrikizumab yielded greater FEV_1_ improvements versus placebo, suggesting that, when targeted to patients with prior exacerbations and evidence of type 2 inflammation, lebrikizumab can be effective [195,196].

The data indicates that selective IL-13 inhibition significantly lowers exacerbation risk in moderate to severe eosinophilic asthma. Still, these findings cannot be generalized to other IL-13 inhibitors like tralokinumab, because binding characteristics differ markedly. In vitro assays show that lebrikizumab’s affinity for IL-13 is approximately 140-fold greater than that of tralokinumab, and IL-13 dissociates more slowly from lebrikizumab than from tralokinumab [197].

## 5. Real-World Evidence for Biologic Therapies in Severe Asthma

While RCTs have established the efficacy and safety of biologic therapies in severe asthma, patients enrolled in RCTs often represent a select population that may not reflect the full spectrum of disease encountered in routine clinical practice. Real-world evidence (RWE) studies have become increasingly important in validating the generalizability of trial findings and assessing the effectiveness of these therapies across more diverse patient groups, including those with multiple comorbidities, varying adherence, and more complex phenotypes. One notable example is the REALITI-A study, a prospective, international observational cohort that evaluated mepolizumab in patients with severe eosinophilic asthma [198]. This study confirmed significant reductions in exacerbation rates and oral corticosteroid use, as well as improvements in asthma control, mirroring outcomes from pivotal RCTs. Similarly, the CHRONICLE study [199] provided key insights into the real-world use of benralizumab, mepolizumab, and reslizumab in the United States, demonstrating substantial reductions in exacerbations and OCS dependence among biologic-treated patients.

These and other RWE publications highlight that biologic therapies remain effective and well tolerated even in broader, less-selected asthma populations. The findings support the utility of blood eosinophil count, exacerbation history, and other biomarkers as predictors of response, not only in clinical trials but also in daily practice. Incorporating real-world evidence thus strengthens the case for personalized and biomarker-driven use of biologics in severe asthma.

## 6. Methodology

**Figure 3:**
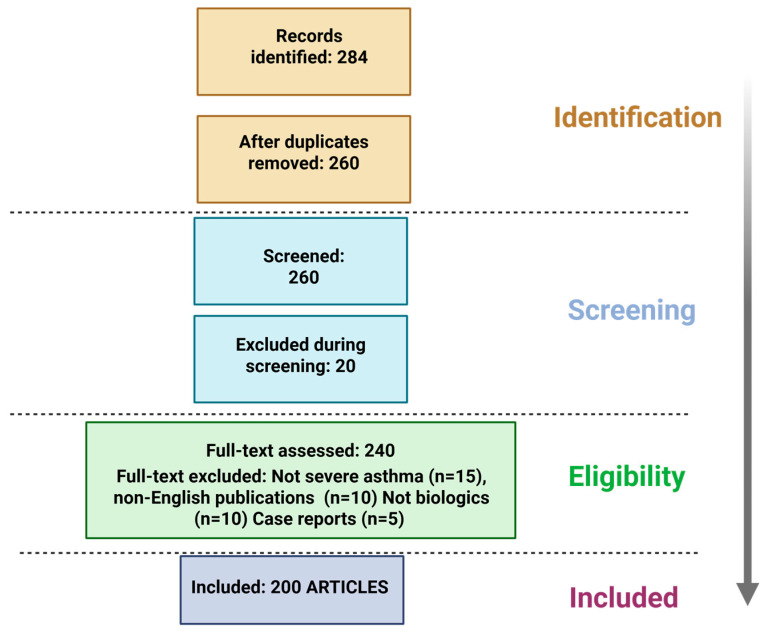
PRISMA flow diagram of study selection process.

A comprehensive literature search was performed in accordance with PRISMA guidelines, utilizing PubMed, Google Scholar, and ScienceDirect as primary databases. Emphasis was placed on articles published within the past five years to capture the most current insights into asthma immunobiology and biologic therapies, although foundational studies predating this window were included when they provided essential mechanistic or clinical context. Search terms combined core disease descriptors—such as “asthma”, “airway inflammation”, “cytokines”, “type 2 inflammation”, and “asthma exacerbations”—with names of established and investigational biologics (e.g., “omalizumab”, “mepolizumab”, “benralizumab”, “depemokimab”, “itepekimab”, “dupilumab”, “tralokinumab”, “lebrikizumab”, “tezepelumab”, “stapokibart”) as well as key upstream mediators (“IL-4”, “IL-5”, “IL-13”, “IL-33”, “TSLP”). In addition, combinations of “biomarkers”, “eosinophils”, “IgE”, “FeNO”, “clinical trials”, and “mechanism of action” were employed to ensure comprehensive coverage of preclinical and clinical data. Inclusion criteria required studies to address one or more of the following: asthma pathophysiology (particularly pro-/anti-inflammatory dynamics and the role of allergic sensitization), cytokine-mediated mechanisms, exacerbation biology, biomarker-driven phenotyping, or the efficacy, safety, and pharmacokinetics of biologic agents. Review articles, randomized controlled trials, observational cohorts, and mechanistic investigations were considered, provided they offered substantive data on biologic therapy selection or outcomes. Exclusion criteria ruled out non-peer-reviewed content, case reports with fewer than ten participants, non-English publications, and studies lacking relevance to severe or poorly controlled asthma. Initial identification via Google Scholar was followed by targeted retrieval of full texts from PubMed and ScienceDirect, with cross-referencing to capture any additional pertinent publications (Figure 3).

## 7. Conclusions

Asthma inherent heterogeneity has long challenged clinicians, as traditional anti-inflammatory and bronchodilator regimens fail to address the diverse immunological pathways underpinning severe disease. The advent of biologic therapies marks a paradigm shift, enabling direct interception of key molecular drivers, namely IgE, IL-5, IL-4, IL-13, IL-33, and TSLP, that orchestrate airway inflammation, remodeling, and exacerbations. By leveraging biomarkers such as blood and sputum eosinophils, serum IgE, and fractional exhaled nitric oxide, clinicians can more precisely identify patients whose disease is driven by type 2 cytokines or alarmin pathways, thereby optimizing the selection of agents most likely to yield clinical benefit.

Anti-IgE therapy established proof of concept, demonstrating reduced exacerbation rates and improved lung function in allergic asthmatics. Subsequent anti-IL-5 agents (mepolizumab, benralizumab, reslizumab, and now depemokimab) extend this success to eosinophilic phenotypes, with trials confirming significant steroid-sparing effects and fewer hospitalizations. The longer half-life of newer constructs like depemokimab supports extended dosing intervals, easing treatment burden. Targeting upstream alarmins—through agents such as itepekimab (anti-IL-33) and anti-TSLP antibodies—shows promise in mitigating tissue eosinophilia and broad inflammatory cascades before they fully develop. Meanwhile, dual IL-4Rα blockers (dupilumab, stapokibart) and IL-13-specific inhibitors (tralokinumab, lebrikizumab) highlight the critical interplay between IL-4 and IL-13 in sustaining type 2 inflammation, though their ultimate efficacy hinges on careful patient stratification via biomarker profiles.

Collectively, these biologics illustrate how elucidating asthma’s molecular heterogeneity enables tailored interventions that not only improve symptom control and lung function but also reduce exacerbations and healthcare utilization. Future research should focus on head-to-head comparisons, refinement of predictive biomarkers, and exploration of combination strategies to achieve durable remission. As our understanding of airway immunobiology deepens, integrating novel biologic agents into treatment algorithms will be essential to deliver truly personalized, mechanism-based care for patients with severe asthma.

## Figures and Tables

**Table 1:** The cytokines in asthma.

Cytokine	Primary Targets	Role in Asthma Pathogenesis	Ref.
IL-4	Th_2_ lymphocytes, B cells	Orchestrates Th_2_ polarization and drives IgE class switching, fueling allergic sensitization.	[47]
IL-5	Eosinophils	Promotes eosinophil maturation, survival and trafficking to the airways, a hallmark of eosinophilic asthma.	[48]
IL-6	T cells, B cells, macrophages	Acts as an acute-phase mediator; elevated in severe exacerbations and linked to systemic inflammation.	[49]
IL-9	Mast cells	Enhances mast cell proliferation and stimulates mucus gland secretion, contributing to airway obstruction.	[50]
IL-10	Regulatory T cells, macrophages	Dampens excessive inflammation by suppressing pro-inflammatory cytokines, helping maintain immune balance.	[51]
IL-13	Airway epithelial cells, goblet cells	Triggers goblet-cell hyperplasia and mucus overproduction; increases airway hyperresponsiveness and contributes to airway remodeling.	[52]
IL-17	Neutrophils	Recruits and activates neutrophils, driving neutrophilic inflammation seen in severe, steroid-resistant asthma.	[53,54]
IL-25	Dendritic cells, ILC2	Potentiates innate type-2 responses by activating ILC2s and priming DCs for Th_2_ cytokine release.	[55]
IL-33	ILC2, mast cells, eosinophils	Functions as an alarmin; amplifies type-2 inflammation and remodels airway tissue via multiple effector cells.	[52]
TGF-β	Fibroblasts, airway smooth muscle	Drives subepithelial fibrosis and smooth muscle proliferation, key processes in airway remodeling.	[56]
TNF-α	Neutrophils, eosinophils, macrophages	Potent pro-inflammatory mediator that recruits innate cells and exacerbates airway inflammation.	[57]
IFN-γ	Th_1_ lymphocytes, macrophages	Exerts a modulatory role by counterbalancing Th_2_ activity but may also contribute to chronic inflammation.	[58]
TSLP	Dendritic cells, Group 2 innate lymphoid cells (ILC2), eosinophils, basophils	Functions as an epithelial cell-derived alarmin; activates dendritic cells and ILC2s, promoting type 2 inflammation and bridging innate and adaptive immune responses; key initiator of airway inflammation and a therapeutic target for tezepelumab	[59]

**Table 2:** Clinical studies evaluating the efficacy of omalizumab in asthma.

Study/Trial Name	Study Type	Key Findings	Reference
Systematic Review	25 Randomized Controlled Trials	Omalizumab led to ~25% reduction in asthma exacerbations and reduced need for ICS use.	[105]
INNOVATE Study	Randomized Controlled Trial	In patients with severe asthma on high-dose ICS + LABA, omalizumab significantly reduced exacerbations.	[106]
EXTRA Trial	Randomized Controlled Trial	Omalizumab treatment resulted in a ~25% decrease in asthma exacerbation rates.	[92]
Prospective Study	Observational (806 patients)	87% of patients showed improvement in asthma symptoms and lung function.	[107]

**Table 3:** Anti-IL-5 and IL-5Rα biologics approved for severe eosinophilic asthma.

Biologic	Mechanism of Action	Approved Age	Eosinophil Threshold	Dosage	Key Efficacy Results	Notable Trials
Mepolizumab (Nucala)	Anti-IL-5; blocks IL-5 from binding to IL-5Rα on eosinophils	≥6 years	≥150 cells/μL	100 mg SC every 4 weeks	53% reduction in exacerbations (MENSA); OCS reduction (SIRIUS); QoL improvement (MUSCA)	MENSA, SIRIUS, MUSCA [117]
Reslizumab (Cinqair)	Anti-IL-5; binds circulating IL-5 to prevent receptor engagement	≥18 years	>400 cells/μL for best response	IV infusion (dose vary by weight) every 4 weeks	92% eosinophil reduction; 50% and 41% reduction in exacerbations; 0.160 L FEV_1_ improvement	Study 3082 (Phase 3) Study 3083 (Phase 3) 3084 (Phase 3); Study 3085 (Phase 3) [118]; REALITI-A real-world cohort [119]
Benralizumab (Fasenra)	Anti-IL-5Rα; induces ADCC leading to eosinophil apoptosis	≥12 years	>300 cells/μL	30 mg SC every 4 weeks (3 doses), then every 8 weeks	51% reduction in exacerbations (SIROCCO, CALIMA); 75% reduction in oral corticosteroid use (ZONDA); FEV_1_ improvement	SIROCCO, CALIMA, ZEPHYR 2, ZONDA [120]

**Table 5:** Key clinical studies evaluating reslizumab in severe eosinophilic asthma.

Effect	Study Type	Key Findings	Reference
Eosinophil Reduction Study (Phase 3)	Clinical Trial	Reslizumab reduced eosinophil levels by 92%, compared to 21% in placebo group.	[132]
Exacerbation Reduction Trial 1 (Phase 3)	RCT, Double-Blind, Placebo-Controlled	Demonstrated a 50% decrease in asthma exacerbations in reslizumab-treated patients.	[133]
Exacerbation Reduction Trial 2 (Phase 3)	RCT, Double-Blind, Placebo-Controlled	Showed a 41% reduction in asthma exacerbation rate with reslizumab use.	[134]
Lung Function Improvement Study (Phase 3)	RCT	Found a 0.160 L greater improvement in FEV_1_ in the treatment group vs. placebo.	[135]
Regulatory Summary	Clinical Overview	Approved as add-on therapy for adults (≥18 years) with severe eosinophilic asthma.	[131]
Stratified Eosinophil Response Study (Phase 3)	Clinical Trial	Enhanced lung function seen only in patients with eosinophil counts > 400 cells/μL; no FEV_1_ improvement in lower counts.	[136]

**Table 7:** Clinical trials evaluating dupilumab across multiple type 2 inflammatory diseases.

Disease Area	Study Name (Clinical Trial ID)	Participant Criteria	Dosage Scheme	Study Duration	Primary Outcome
Atopic Dermatitis	SOLO-1 (Phase 3; NCT02277743)	Adults (≥18 yrs), moderate to severe cases, *n* = 671	300 mg weekly/biweekly; placebo	16 weeks	Investigator’s Global Assessment (IGA) score 0/1 with ≥2-point drop [149]
	SOLO-2 (Phase 3; NCT02277769)	Adults (≥18 yrs), moderate to severe, *n* = 708	300 mg weekly/biweekly; placebo	16 weeks	IGA score 0/1 and ≥2-point drop [149]
	CHRONOS (Phase 3; (NCT02260986)	Adults (≥18 yrs), moderate to severe, *n* = 740	300 mg weekly/biweekly; placebo	52 weeks	IGA score 0/1 and ≥2-point drop [149]
	LIBERTY AD ADOL (NCT03054428)	Adolescents 12–17 yrs, moderate to severe	<60 kg: 200 mg biweekly; ≥60 kg: 300 mg biweekly; placebo	16 weeks	IGA score 0/1 and ≥2-point drop [149]
	LIBERTY AD PEDS (Phase 3; NCT03345914)	Children 6–11 yrs, severe	<30 kg: 100 mg biweekly; ≥30 kg: 200 mg biweekly or 300 mg every 4 weeks; placebo	16 weeks	IGA score 0/1 [150]
	LIBERTY AD PRESCHOOL (Phase 3; NCT03346434)	Children 6 months–5 years, moderate to severe	5–15 kg: 200 mg every 4 weeks; 15–30 kg: 300 mg every 4 weeks; placebo	16 weeks	IGA score 0/1 [150]
Asthma	LIBERTY ASTHMA QUEST (Phase 3; NCT02414854)	Patients ≥ 12 yrs, moderate to severe, *n* = 1902	200 mg or 300 mg biweekly; placebo	52 weeks	Exacerbation frequency; FEV_1_ change [151]
	LIBERTY ASTHMA VOYAGE (Phase 3; NCT02948959)	Children 6–11 yrs, moderate to severe, *n* = 408	<30 kg: 100 mg biweekly; ≥30 kg: 200 mg biweekly; placebo	52 weeks	Annualized exacerbation rate [152].
hronic Rhinosinusitis with Nasal Polyps	NP SINUS-24 (Phase 3; NCT02912468)	Adults (≥18 yrs), severe symptoms, *n* = 276	300 mg biweekly; placebo	24 weeks	Change in nasal congestion, polyp score, and CT findings [153]
	NP SINUS-52 (Phase 3; NCT02898454)	Adults (≥18 yrs), severe symptoms, *n* = 448	300 mg biweekly for 24 weeks, then monthly; placebo	52 weeks	Change in nasal polyps, congestion, CT at 24 weeks [153]
Eosinophilic Esophagitis	Parts A, B, C (Phase 3; NCT03633617)	Adults ≥ 12 yrs	300 mg weekly or biweekly; placebo	24–28 weeks	Histological remission (≤6 eos/hpf), symptom reduction [154].
	EoE KIDS (Phase 3; NCT04394351)	Children 1–11 yrs	<30 kg: 200 mg biweekly; 30–60 kg: 300 mg biweekly; placebo	16–52 weeks	Histologic response defined as ≤6 eosinophils per hpf [155].

**Table 8:** Summary of clinical trials evaluating tralokinumab in patients with moderate-to-severe asthma.

Study (ID)	Japanese LTS Phase 3	MESOS Phase 2	TROPOS Phase 3	STRATOS 2 Phase 3	STRATOS 1 Phase 3
Subjects Enrolled	28	79	140	856	1207
Lead-in Phase	Up to 2 weeks	—	2 weeks	4–6 weeks	4–6 weeks
Active Treatment Span	52 weeks	12 weeks	40 weeks	52 weeks	52 weeks
Post-treatment Follow-up	14 weeks	14 weeks	14 weeks	20 weeks	20 weeks
Enrollment Criteria	Japanese adults with asthma not controlled on ICS + LABA	Asthmatics uncontrolled on ICS alone	Asthmatics requiring maintenance OCS + ICS/LABA	Adults with asthma inadequately managed on ICS/LABA	Adults with asthma inadequately managed on ICS/LABA
Investigational Dose	300 mg SC every 2 weeks	300 mg SC every 2 weeks	300 mg SC every 2 weeks	300 mg SC every 2 weeks	300 mg SC every 2 weeks or 300 mg SC every 4 weeks
Comparator Arm	None (open-label)	Placebo SC every 2 weeks	Placebo SC every 2 weeks	Placebo SC every 2 weeks	Placebo SC every 4 weeks
Primary Aim	Evaluate long-term tolerability and safety in Japanese patients	Examine impact on eosinophil-driven airway inflammation and remodeling	Determine whether tralokinumab allows reduction in maintenance OCS dose	Confirm efficacy/safety in biomarker-positive subgroup	Assess efficacy/safety in overall population and identify biomarker-positive subgroup
Primary Endpoint	Rate and severity of adverse events (AEs) [190]	Change in submucosal eosinophil density (cells/mm^2^) after 12 weeks [190]	Percent change in average daily OCS dosage at week 40 [191]	Annualized asthma exacerbation rate (AAER) through week 52 [192]	AAER through week 52 [192]

## Data Availability

No new data were created or analyzed in this study.
